# Editorial: Pharmaceutical biomaterials

**DOI:** 10.3389/fbioe.2025.1661817

**Published:** 2025-08-05

**Authors:** Donghan Shao, Yuqin Ma, Yunhui Li, Qingming Xu, Leijiao Li, Wenliang Li

**Affiliations:** ^1^ School of Chemistry and Environmental Engineering, Changchun University of Science and Technology, Changchun, China; ^2^ Zhongshan Institute of Changchun University of Science and Technology, Zhongshan, China

**Keywords:** pharmaceutical biomaterials, smart drug delivery, engineered biomaterials, functional biomaterials, tumor immunity

## 1 Introduction

Biomaterial-pharmaceutical convergence advances smart drug delivery. Engineered biomaterials enable precise therapies via biocompatibility/tunability. This Research Topic showcases multifunctional systems through 32 contributions, spanning composites to translational research for clinical translation.

## 2 Biomimetic structures guiding regeneration

Functional biomaterials integrated into multi-scale bionic designs enable synergistic control of angiogenesis, antibacterial activity, osteogenesis and so on. Wang’s 3D-printed silicate scaffold integrates calcium sulfate-Cu^2+^ delivery: rapid calcium sulfate dissolution initiates osteogenesis, sustained silicate degradation maintains support, and Cu^2+^ release enables antimicrobial/angiogenic niches, achieving spatiotemporal bone repair synchronization (Gao et al.). The transform inert materials into dynamic regenerative platforms through structural programming, proving structural precision enables *in situ* tissue rebirth.

## 3 Nanotechnology reprogramming tumor immunity

Nanotechnological remodeling of the immunosuppressive tumor microenvironment (TME) represents a paradigm-shifting therapeutic frontier. Tian’s Mn nanozyme acts as a metabolic surgeon in acidic TME: dual peroxidase/catalase activities decompose H_2_O_2_ into ∙OH/O_2_, inducing ROS ablation while alleviating hypoxia. Liberated Mn^2+^ blocks PD-L1, synergizing with photothermal immunogenic cell death (ICD) to suppress tumors (Yang et al.). Chen’ group reviewed some related studies on the hyperthermia-enhanced checkpoint blockade effect (Xie et al.). Nanomaterials evolve from passive carriers to active TME builders, reshaping the tumor immune environment.

## 4 Smart drug delivery overcoming pathological barriers

Intelligent nanomaterials enable on-demand drug release matched to disease rhythms via stimuli response, overcoming pathological barriers. Wu et al. developed NIR-triggered Au-Ag-PDA@MSCM nanosystems inducing local hyperthermia-mediated ferroptosis via Acsl4 upregulation, achieving triple precision: spatial (MSCM homing), temporal (photoactivation), and metabolic (Acsl4 hijacking) control. Zheng et al. designed an injectable dual-carrier system: ① Van-loaded Gel combats infection, ② exposed PLGA MPs release DGEA osteogenic peptide during transition, ③ new bone regenerates. Intelligent systems transform into active agents, remodeling microenvironments for full-cycle disease modulation.

## 5 Conclusion

Collectively, these advances mark a transformative shift toward real-time interactive therapeutic platforms. By converging materials science, biology, and engineering, the field delivers clinically viable solutions that dynamically intercept disease progression, enabling adaptive precision medicine.

